# Some General Remarks on Fractures and Their Treatment, Tending to Illustrate the Causes of Failure of Union of Those Related by A. Carlisle, Esq.

**Published:** 1801-11

**Authors:** G. Wilkinson

**Affiliations:** C. M. S. of London, Member of the Royal College of Surgeons, and Honorary Member of the Chirurgo-Physical Society of Edinburgh, &c.


### *Some general Remarks on Fractures and their Treatment, tending to illustrate the Causes of Failure of Union of those related by* A. Carlisle, Esq.

By G. Wilkinson, C. M. S. *of London, Member of the Royal College of Surgeons, and Honorary Member of the Chirurgo-Physical Society of Edinburgh, &c.*

---

### To Dr. BRADLEY.

Sir,

SUCH cafes of failure of union in fimple fractures, as related by the ingenious and indefatigable Mr. Carlifle,* are luckily not very common; admitting however the truth of this remark fo far as it relates to recorded cafes of this fort, yet for any thing we know to the contrary, they may have, and do oftener occur than we are acquainted with. It is rare indeed, and requires fome degree of firmnefs of mind in men to relate unfuccefsful cafes, and confefs errors into which they have been inadvertently led; to human nature this feems too mortifying. Hence I have been led to efteem and refpect highly thofe perfons who frankly and candidly acknowledge their own imperfections, and have in view as their object, the correction and improvement of an art, that in itfelf is ftill allowed to be but imperfect and incomplete.

With thefe reflections I have been impelled, on the perufal of Mr. C's ufeful obfervations and inquiries, to offer my fentiments on what may appear to be the general caufes of retardation, or want of union in fimple fractures, noticing at the fame time the cafes he has recorded; and fhall alfo indulge myfelf with fome remarks on the common routine of practice, comparing this with what has fallen under my own obfervation, and with that which I have ufually adopted.

If I may be permitted to point out the general caufes of retardation, or want of union in fimple fractures, and which in many refpects may be applied to compound, they will refolve themfelves into two diftinctions, viz. general and local, or what may be termed, conftitutional and mechanical.

The general caufes may proceed from fome conftitutional difeafes, as rickets, *mollities ossium*, fcrophula, fcurvy, and in fome cafes of pregnancy; it may likewife be induced by a debi-

---

* Vide Medical and Phyfical Journal, No. XXXI.

litated ftate of the fyftem occafioned by fome difeafe antecedent to the accident, or brought on by too profufe evacuations, low diet, and depreffion of mental energy.

Mechanically or locally, it has fometimes happened that a portion of mufcular flefh, tendon, or ligament intervening between the ends of the bones, has prevented them from uniting; a faulty pofition of the limb by preventing the bones from coming into clofe contact, and inducing œdema, which retards the venous circulation, as was ufually the cafe in the old mode of practice, by keeping the limb in a ftate of extenfion; a detached piece of bone or fplinter intervening between the ends of the fracture, is well known to prevent union, and what does not very often happen in fimple fractures, although in fome inftances it has in an effufion or extravafation of blood from the rupture of blood-veffels remaining long in contact, will produce the fame effect; to fome one or more of thefe caufes, aided by the conftitutional debility that enfued from the mode of treatment, are to be afcribed the want of union in Mr. C's patients, which we fhall notice more particularly hereafter.

In the ufual mode of treatment generally adopted of fractures, it is not uncommon in the very firft onfet, to bleed largely from the fyftem, open the belly very freely, confine the patient to a very low diet, and apply warm fomentations, poultices, &c. This practice although in fome inftances proper, and even judicious, will in other cafes prove hurtful, and diminifhes the vital energy, depreffes the ftrength, relaxes the fyftem, and favours the continuation of the tumefaction of the limb. By thefe means, which through over officioufnefs are too frequently applied to prevent fever and inflammation, much hurt is often done, as the very thing is prevented which would produce a fpeedy and healthy union of the parts, viz. the adhefive inflammation. If we add to thefe the continuation of low diet, and keeping the patient in bed in an uniform pofture, affifted by copious perfpiration which often enfues, the debility is of courfe increafed. Independent of any peculiarity or unfavorablenefs of the injury, or of the conftitution of the patient, it cannot be doubted but a long and tedious cure will be the confequence.

I have already concurred in opinion with Mr. C. that cafes of defective offification after fimple fractures are uncommon,* and allow

* A cafe of this fort fell under my own obfervation fourteen years ago. The wife of P. Wright, fhoemaker of this place, upwards of feventy years of age, fell off a ftool not more than thirteen or fourteen inches in height, and fractured her left thigh; this was neither difcovered nor fufpected till more allow they muſt be diſtreſſing to the patient and ſurgeon, but that "there is no decided inference to be drawn from theſe caſes, as to the certain cauſes of ſuch misfortunes," which are his words, is what I very much doubt; for this reaſon I have been been led, which I ſhould not otherwiſe have done, to glance briefly by a conciſe view, at what experience and obſervation have acknowledged as the general cauſes of a want of union, and ſhall now attempt to aſcertain, or account for what may be deemed the true cauſes of the failure of thoſe he has related.

In the hiſtories he has given us, from his own confeſſion, it evidently appears that the depleting ſyſtem, or what may be termed the antiphlogiſtic plan, ſeems to have been carried to too great an exceſs; the evacuations were extenſive, the diet low and ſlender, and although this is not mentioned in the caſe of the ſailor, yet the abuſe of ſpirits, conſtant uſe of tobacco, &c. by inducing eventually an *aſthenic* ſtate of the ſyſtem, from their injuring the ſtomach and digeſtive organs, the difficulty with which fractures of the thigh are retained in coaptation, his being on ſhipboard, its liableneſs to diſtortion from motion in ſuch a ſituation, if it were ever reduced at all, even according to the *common routine*; and if to theſe we add the diſpoſition to *ſcorbutic diatheſis*, which prevails more or leſs in ſhips of war in ſome degree, with the miſplaced poſition of the bones, which overlapped each other two inches for a period of fifteen months, and the circumlocution, if I may be allowed the expreſſion, of the oſſific proceſs, which in diſtortions, even in the beſt conſtitutions, takes up perhaps more than double the time to unite them, we ſhall not I apprehend be at a loſs to account for a ſort of diſpoſition, or *acquired habit*, that unfortunately in this very ſingular caſe, which from the failure of the operation performed upon it, as Mr. C. obſerves, "ſhewed ſomething, (and indeed beyond a doubt,) like a conſtitutional deficiency in the oſſific proceſs."

---

more than three weeks had elapſed, when on being ſent for promiſcuouſly to ſee her, and ſhe complaining of an inability of moving her thigh, I diſcovered by the diſtortion and jarring of the bones, that it had been broke from that time. No appearance of inflammation ſeemed to exiſt, very little pain except on motion, and little or no tumefaction had occurred; and although I reduced it with the greateſt eaſe, and placed it in a proper poſition, yet after three months had paſſed over, no union had taken place. She then threw away the ſplints, and would not ſuffer them to remain any longer; and although ſhe lived about twelve months after this, the fracture ſtill remained looſe. This woman was infirm, much extenuated, and her pulſe low and languid. She could not be prevailed upon to take any ſort of medicine whatever.

 Conſidering

Confidering this however as an uncommon and almoft *unique* cafe, it certainly was equally meritorious for the man to undergo as the furgeon* to attempt an operation of fome difficulty by diffecting down, and fawing off the ends of the bones, with a fair and I may fay rational view, of affording, perhaps, the only chance of an union.† Mr. C. in the courfe of his narrative of this cafe, has faid, after fifteen months had elapfed: "The man was athletic, of a dark complexion, &c." If by this he means robuft or ftrong, it feems not a little ftrange he might appear fo, as we often fee many hard drinkers who are not fhrunk in their mufcles, yet in their nervous fyftem are much debilitated; Mr. C. however obferves, when defcribing the general habit, or conftitution of thefe three patients, the fubject of his hiftories, that,

"In all thefe men the vafcular fyftem appeared fluggifh, their pulfes were flow, and the characteriftics of inflammatory difpofition were wanting."

Now whether this ftate of the fyftem which Mr. C. has defcribed, was natural or peculiar to thefe patients prior to the accidents, or it was their actual fituation at the time they laboured under their indifpofitions from the mode of treatment, feems not afcertained; if it was natural or peculiar, the antiphlogiftic plan and low diet was not only unneceffary but hurtful; if otherwife, the ftate of the fyftem he has defcribed muft have been induced by the treatment itfelf. In the cafe of the houfe carpenter, where the fracture was in the middle of the os brachii, it is mentioned that his arm had *always* hung low in the fling, "the fore arm and hand were conftantly œdematous, and the upper arm had ftretched full two inches in length, fo that the ends of the fracture were feparated to this diftance."

The pofition of this arm, which Mr. C. fays had *always* hung low in the fling, and during the earlier period, was kept *remarkably straight*, and which he confiders "*must have been in a favorable position for union*, to me appears ftrange, as it is a pofture altogether unufual, not to fay unnatural, and as it probably tended to produce, if not to encourage, the œdamatous fwelling of the fore arm and hand, thereby proving unfavorable to the venous circulation in the limb, accounts, I think

* Some years ago, that accurate anatomift and expert furgeon, Mr. Lynn, fenior Surgeon of the Weftminfter Hofpital, performed an operation of this fort on the fractured thigh of a patient; but whether this may be the perfon alluded to by Mr. C. I am uncertain.

† The fame operation has alfo been performed by Mr. White, of Manchefter. Vide Cafes of Surgery by C. White, &c.

think, *decidedly*, together with the extenſive lowering plan, for a want of union.* In the third caſe, which is that of the ſoldier, who had his "tibia broken and fibula diſlocated," he obſerves that the lowering plan was carried to the full extent, and "after ten months the broken bone was looſe," but admits that a "piece of ſplinter appeared to be intervening," yet allows as "very probable, that this man's limb became firmer," although he "never heard of his fate," is what moſt likely would take place, when the ſplinter was removed.

The remarks which I have already made on the common routine of treatment, may be equally applied to fractures in general, whether ſimple or compound. Inſtead of evacuating largely by bleeding from the ſyſtem, except in particular inſtances, it would be more proper to do this by leeches applied to the parts, and as a ſubſtitute for fomentations and warm poultices, which in ſimple fractures tend to keep up the tumefaction or ſwelling of the limb, and even in compound ones, bring on too large and extenſive ſuppuration, and not unfrequently profuſe hæmorrhagies; it would be much more adviſeable to uſe liberally as applications, ſedatives, ſuch as acetum. c. ſal. ammon. crud. aq. veget. min. c. ſp. vin. camph. and very light and ſimple dreſſings in compound caſes, and theſe removing as ſeldom as poſſible, unleſs when the diſcharge renders it abſolutely neceſſary.

To ſay that tailed bandages ought to be uſed on all occaſions, whether in ſimple or compound fractures, would be ſuperfluous, as this I truſt, in this enlightened age, is now adopted by all regular practitioners. In every caſe, whether ſimple or compound, reduction of the fracture ought to take place as ſoon as poſſible; I have frequently ſucceeded many hours, and even after a whole day has elapſed after the accident, where conſiderable ſwelling and tumefaction had begun. Cloths well wet with vinegar and cold water, or with cold water alone, where vinegar is not to be had, poured at ſome height upon the part or limb, or even ice, might be applied with ſucceſs, to diminiſh the ſwelling. This mode of treatment, which I am warranted from experience to enforce, will in general enable us to proceed to the immediate and prompt reduction of the fracture; for while I have employed my aſſiſtants in uſing theſe means, I have been occupied in making ready the neceſſary apparatus for reduction. As the limb ſhould be placed, the firſt

* I would aſk Mr. C. why the ſtraight poſition of the arm, and its hanging low in the ſling, is favorable to an union? Does he ſpeak from theory, or from experience, or facts that have fallen under his obſervation?

firft thing we do, in an eafy pofition, to relax the tenfion of the mufcles, very little extenfion is feldom required, and the fooner the reduction takes place the better; for when this has been prevented or neglected, the great length of time that is ufually taken up before the tumefaction and inflammation which are going on fubfide, the fhortening of the mufcles, and union that in fome degree more or lefs enfues, the diftortion ftill remaining, all thefe, together with the extreme averfion of the patient to being put to pain, and the force which is neceffary to be applied to its reduction being liable to lacerate and tear the mufcles; the whole of thefe confidered, need not caufe us to wonder a diftorted limb will ufually be the cafe, confequently a long and tedious cure.

It is now generally allowed, and I believe univerfally admitted, that fractures of every fort, whether fimple or compound, in private practice, and that generally in the country, have terminated ufually more favourably and fuccefsfully than in public hofpitals, fhips of war, and in naval and military hofpitals, even under the moft judicious treatment; and although the caufe of failure in thefe inftances has been principally attributed to a vitiated and contaminated atmofphere, ufually prevailing in thefe places, and which it is not always in our power to prevent; yet when we confider the ftrict, and for the moft part, lowering fyftematic plan of diet that is ufually adopted in hofpitals, and the general treatment, which for the moft part is of a depleting nature, aided as it were by the depreffion of ftrength that often refults from the energy of the mind being weakened by the abfence of the patient's friends, we fhall be lefs at a lofs to develope thefe as fome of the caufes of retardation and want of fuccefs.

To the lowering plan of treatment not being fo ftrictly purfued in private practice, by the patient's friends indulging him clandeftinely in a more free and liberal diet, which is ufually the cafe, it is probably owing that our ill-judged (though well-meant) intentions of depreffing his ftrength, has been counteracted, and although fuch proceedings have been often productive of evil, yet it has been probably owing to this, as one principal caufe, that fractures in general, in private practice, have terminated more favorably than in public hofpitals, &c. where fuch deviations from the regular routine could not have been well effected.

By fudden and long continued abftraction of the ftimulus of nourifhing diet, the fyftem will moft affuredly fuffer; of this fact I have been convinced from what has occurred in the early part of my practice, fo that whenever a drunken or intemperate perfon meets with an accident, fuch as fracture, bruife, fprain,

ſprain, or injury from a fall, and even in ſeveral other medical and chirurgical diſeaſes (with ſome exceptions that may occur) I have found it even neceſſary, and not altogether ſafe; to detract entirely from their intemperate habits. For this hint I feel myſelf indebted to the late Profeſſor Monro, who has given us ſeveral caſes of the ſuccefsful indulgencies of bad habits;* and the ingenious Dr. Kirkland has noticed the ſame.† A drunken taylor fractured the tibia and fibula of his left leg; the bone protruded through the ſkin two inches, it was however ſoon reduced; in this caſe tin ſplints, a very uſeful late diſcovery in compound fractures, were uſed; the wound was dreſſed but very ſeldom, and although I dreaded a tedious cure from the nature of the caſe, and his well-known intemperance, yet by allowing him a quart of ale per diem, to which he added, by his own confeſſion, two glaſſes of gin, being about a half or one-third of his common allowance; yet, ſtrange to ſay, when three weeks were expired he ſewed light work in bed, and after ſix or ſeven weeks had elapſed walked to the workſhop, more than three hundred yards diſtance, by means of a ſtaff, the bones being completely united and the wound healed; no fomentations or poultices were uſed here, nor did he require bleeding.

In another caſe of ſimple fracture of both bones of the right leg, in a ſtout man of an intemperate habit, and who indulged himſelf with tolerable freedom, it was united in leſs than ſix weeks, he having at that time got about on crutches. This fracture was ſomewhat oblique, though not reduced till the day after the accident, as I was from home. He was treated in the ſame way as the former.

Many other inſtances I could relate of the ſame, but theſe I truſt may ſuffice as deviations from the uſual routine.

To illuſtrate in ſome meaſure the abſtraction of bad habits, in oppoſition to thoſe already related, it may not be altogether unneceſſary to remark, that in a caſe of fractured tibia in a gentleman, and one of the os humeri in an elderly woman, both of which had been accuſtomed to indulge freely, I took upon me to reſtrain them entirely from their bad cuſtom. I however found a great difference, for after ſix weeks had elapſed they were languid and depreſſed, their pulſes ſluggiſh, and appetite depraved. Although I afterwards permitted them to indulge ſomewhat in their habits, which I have no doubt they did, yet ten weeks had paſſed over before the ſtrength was reſtored,

* Vide Edinb. Medical Eſſays, Vol. V. VI.

† Medical Surgery, Vol. I. II.

reftored, and the cure firmly eftablifhed.—Thefe inftances which I have related, and which came under my immediate infpection, are not advanced with a view to recommend free indulgences as a practice to be adopted generally, as I am fully aware, where free licence is given, it is too often carried to excefs; they are adduced only as deviations from the ufual routine, and tend to demonftrate the imperfection of the lowering plan, and the neceffity, in fome meafure, of conniving at habits, though in general hurtful, which cannot at all times be fafely retrenched.

In violent pain, which is ufually attendant, in a greater or leffer degree, in almoft every fort of fracture, fprains, diflocations, bruifes, &c. and which is admitted as the criterion of the adhefive inflammation, or healthy union, that is going on, although it may be, and is in fome cafes, combined with fpafm, I have fometimes bled from the fyftem where a general plethora was obvious; otherwife, when this was not the cafe, topically by leeches. But as the violence of this pain is by no means conftant, and almoft invariably is increafed towards night, no doubt from the acceffion of the natural evening paroxyfm of fever, which commences about fix or feven o'clock in the evening, I have been led particularly from a violent and diftracting pain in my left wrift, or carpus, which I fprained fixteen years ago, and which was aggravated at the period of the acceffion of the natural evening paroxyfm, though pretty eafy in the courfe of the day, to adopt at bed-time the ufe of fudorific opiates; the quantity of the opiate is ufually increafed every night, according to the urgency of the pain, and when coftivenefs fupervenes, an opening medicine is given in the morning. To this mode of treatment, and what I have already noticed as my general practice, am I indebted for the fpeedy and more fuccefsful termination than ufually happens in accidents of this fort. To the objection that may arife from the ufe of opiates becoming habitual, and to the reft and fleep procured by it being not natural, but forced; I reply, that pain is a difeafe, and eafe and fleep procured, even at any rate, is defirable, and facilitates recovery. The difeafe or pain going off, as it does after a certain period in thefe cafes, the opium is no longer neceffary, as the natural reft returns without it.

As a proof of the benefit derivable from this mode of treatment, it may not be altogether ufelefs to mention a ftriking inftance comparatively of its effects:—A hale ftout man, fixty years of age, or thereabouts, remarkably intemperate; and an healthy woman, aged forty, about two years fince, fractured each of them their fibula, at the fame period of time, and nearly ly ly in the fame place, about two inches above the ankle; the man, being plethoric, was bled ad vires, was permitted to ufe occafionally a fmall quantity of grog, which I fuppofe he ufed moderately; he took the anodyne fudorific at bed-time almoft every night on the acceffion of pain, and when this was omitted, which to pleafe his fancy he ufed to do, he found the want of it.

The woman, however, could not be prevailed upon at any rate to take any fort of medicine; and although they were otherwife treated in the fame way, yet, notwithftanding the difparity of their ages, the man got upon his crutches about five weeks after, whereas feven weeks elapfed before the woman did the fame.

As fome of my readers, who are veterans in the chirurgical art, and whofe reputations are firmly eftablifhed by the confpicuous rank they hold in their profeffion, may be led to fuppofe my general remarks are too diffufive, unneceffarily minute, and common placed, and contain nothing but what is already known and made public, yet their differing from the general routine, as may be feen in Mr. C's paper, which is calculated to invite a liberal and free inquiry, will perhaps appear, on due reflection, not altogether unufeful; and when it is confidered that the want of time in fome, inclination and even difpofition to reflection or ftudy in others, may have prevented them from giving themfelves any trouble how they are to act; and, which is too often done, more from imitation than reflection or intuition, their attentive perufal may poffibly be not unattended with fome profit. And as it is for junior practitioners chiefly, whofe minds, principles, and practice, may not be yet formed, that I was impelled to be thus diffufive,—this apology, I truft, will be deemed fufficient.

To Mr. Carlifle, with whom I am by no means perfonally unacquainted, it may appear I have taken too great liberties in my remarks on the defcription of his cafes; but as this to me feemed abfolutely neceffary towards a full and, I truft, not unjuft inveftigation of a fubject that required, from his own free and candid invitation, a fair difcuffion, I hope he will not confider what I have faid as bordering on illiberality. If I have miftaken his meaning, I am open to conviction, and promife, on his convincing me of any imperfections, to retract what may appear to be erroneous.

I am, Sir, your's, &c.

*Sunderland, Sept.* 27, 1801. G. WILKINSON.

